# Smoking-induced gene expression changes in the bronchial airway are reflected in nasal and buccal epithelium

**DOI:** 10.1186/1471-2164-9-259

**Published:** 2008-05-30

**Authors:** Sriram Sridhar, Frank Schembri, Julie Zeskind, Vishal Shah, Adam M Gustafson, Katrina Steiling, Gang Liu, Yves-Martine Dumas, Xiaohui Zhang, Jerome S Brody, Marc E Lenburg, Avrum Spira

**Affiliations:** 1 Pulmonary Center, Boston University School of Medicine, Albany Street, Boston Massachusetts, USA; 2 Pathology Program, Graduate Medical Sciences, Boston University School of Medicine, Albany Street, Boston Massachusetts, USA; 3 Department of Genetics and Genomics, Boston University School of Medicine, Albany Street, Boston, MA, USA; 4 Bioinformatics Program, Boston University School of Engineering, Cummington Street, Boston Massachusetts, USA

## Abstract

**Background:**

Cigarette smoking is a leading cause of preventable death and a significant cause of lung cancer and chronic obstructive pulmonary disease. Prior studies have demonstrated that smoking creates a field of molecular injury throughout the airway epithelium exposed to cigarette smoke. We have previously characterized gene expression in the bronchial epithelium of never smokers and identified the gene expression changes that occur in the mainstem bronchus in response to smoking. In this study, we explored relationships in whole-genome gene expression between extrathorcic (buccal and nasal) and intrathoracic (bronchial) epithelium in healthy current and never smokers.

**Results:**

Using genes that have been previously defined as being expressed in the bronchial airway of never smokers (the "normal airway transcriptome"), we found that bronchial and nasal epithelium from non-smokers were most similar in gene expression when compared to other epithelial and nonepithelial tissues, with several antioxidant, detoxification, and structural genes being highly expressed in both the bronchus and nose. Principle component analysis of previously defined smoking-induced genes from the bronchus suggested that smoking had a similar effect on gene expression in nasal epithelium. Gene set enrichment analysis demonstrated that this set of genes was also highly enriched among the genes most altered by smoking in both nasal and buccal epithelial samples. The expression of several detoxification genes was commonly altered by smoking in all three respiratory epithelial tissues, suggesting a common airway-wide response to tobacco exposure.

**Conclusion:**

Our findings support a relationship between gene expression in extra- and intrathoracic airway epithelial cells and extend the concept of a smoking-induced field of injury to epithelial cells that line the mouth and nose. This relationship could potentially be utilized to develop a non-invasive biomarker for tobacco exposure as well as a non-invasive screening or diagnostic tool providing information about individual susceptibility to smoking-induced lung diseases.

## Background

Approximately 1.3 billion people smoke cigarettes worldwide contributing to almost 5 million preventable deaths per year [1]. Smoking is a significant risk factor for lung cancer, the leading cause of cancer-related death in the United States, and chronic obstructive pulmonary disease (COPD), the fourth leading cause of death overall. Approximately 90% of lung cancer can be attributed to cigarette smoking, with 10–15% of smokers developing this disease [2]. Despite the well-established causal role of cigarette smoke in lung cancer and COPD, the molecular mechanisms by which these diseases arise are poorly understood and there are no tools currently available to determine individual variations in response to smoking.

Previous work has demonstrated that cigarette smoke creates a field of injury in epithelial cells that line the respiratory tract. Several studies have shown that histologically normal large airway epithelial cells of current and former smokers with and without lung cancer display allelic loss [3,4], p53 mutations [5], changes in promoter methylation [6] and increased telomerase activity [7]. Using epithelial cells collected from brushings of the mainstem bronchus at the time of bronchoscopy, we have previously characterized the effect of smoking on the bronchial airway epithelial transcriptome and found that smoking induces expression of genes involved in regulation of oxidant stress, xenobiotic metabolism, and oncogenesis while suppressing those involved in regulation of inflammation and tumor suppression [8]. In addition, we recently developed a profile of bronchial airway gene expression that can distinguish smokers with and without lung cancer and serve as an early diagnostic biomarker for disease [9]. Although these studies of intrathoracic airway epithelium obtained via bronchoscopy have successfully identified candidate biomarkers of smoking-related lung damage, there remains significant impetus to develop biomarkers of these events from tissue obtained via less invasive collection procedures. Use of material from a less invasive collection site would allow for the use of larger cohorts for developing and validating biomarkers of tobacco exposure and susceptibility to tobacco-related disease.

Oral and nasal epithelium are attractive candidate tissues for assaying the host response to tobacco-smoke exposure since, like the bronchial airway, they are exposed to high concentrations of compounds contained within cigarette smoke. We have previously shown that it is feasible to obtain sufficient RNA from buccal mucosa for gene expression analysis [10] despite the high level of RNAses in saliva [11,12]. Few studies have characterized global gene expression in either buccal or nasal mucosa, and none have attempted to establish a link between extra- and intrathoracic airway gene expression changes that occur with smoking. Smith et al. used brush biopsies of buccal mucosa from smokers and non-smokers to obtain RNA for cDNA microarrays and found approximately 100 genes that could distinguish the two groups in training and test sets [13]. While this study provided evidence that buccal gene expression changes with smoking, it did not address the relationship between the gene expression response to tobacco smoke in the mouth and bronchial airways. Using real-time PCR, Spivack et al. found a qualitative relationship between matched buccal mucosa and laser-dissected lung epithelial cell samples across nine carcinogen or oxidant-metabolizing genes in 11 subjects being evaluated for lung cancer [14]. Smoking has also been implicated in the formation of DNA adducts in nasal mucosa [15], and the correlation between adduct formation in bronchial and nasal epithelium has been previously reported [16]. While global gene expression profiling of nasal epithelial brushing has been recently reported in children with asthma [17] and cystic fibrosis [18], there are no studies that address the effects of smoking on global gene expression in nasal epithelium or explore relationships in gene expression between epithelial cell types throughout the respiratory tract in response to cigarette smoke.

In this study, we examined relationships in gene expression between bronchial, nasal, and buccal epithelial cells in current and never smokers. Using previously published microarray data, we evaluated the relationship of buccal, nasal, and bronchial epithelial gene expression in never smokers compared to other epithelial and non-epithelial tissues. We subsequently compared gene expression consequences of smoking in bronchial epithelial cells to those seen in buccal and nasal epithelium. Our results suggest that gene expression changes occurring in bronchial epithelium in response to cigarette smoke are reflected in buccal and nasal epithelium. As a result, we believe that gene expression biomarkers of host response tobacco exposure may ultimately either be applied to or derived from these tissues.

## Results

### Study Population

Twenty five subjects were recruited for nasal and buccal mucosa microarray studies and 14 additional subjects were recruited for real competitive PCR validation experiments on buccal mucosa samples. Demographic data for the microarray study group is presented in Table 1. Demographic data for the real competitive PCR validation group is presented in Additional File 4.

**Table 1:** Patient demographics

	Buccal Microarray (n = 10)			Nasal Microarray (n = 15)		
	
	Smokers n = 5	Never n = 5	P-Value	Smokers n = 7	Never n = 8	P-Value
Sex	1 M, 4 F	2 M, 3 F	p = 0.42	6 M, 1 F	6 M, 2 F	p = 0.58
Age	36 (+/- 8)	31 (+/- 9)	p = 0.36	47 (+/- 12)	43 (+/- 18)	p = 0.46
Race	3 CAU, 2 AFA	2 CAU, 3 AFA	p = 0.40	3 CAU, 3 AFA, 1 HIS	5 CAU, 2 AFA, 1 HIS	p = 0.73

Demographic data for patient samples used in the microarray studies (n = 25). Clinical information including sex, age, and race are included (CAU – Caucasian, AFA – African American, HIS – Hispanic). Statistical comparisons were performed between smokers vs. nonsmokers within each tissue type. P-values for sex and race were determined by Fisher exact test.

### Relationship of gene expression profiles between intrathoracic and extrathoracic airway epithelium in never smokers

**Figure 1:**
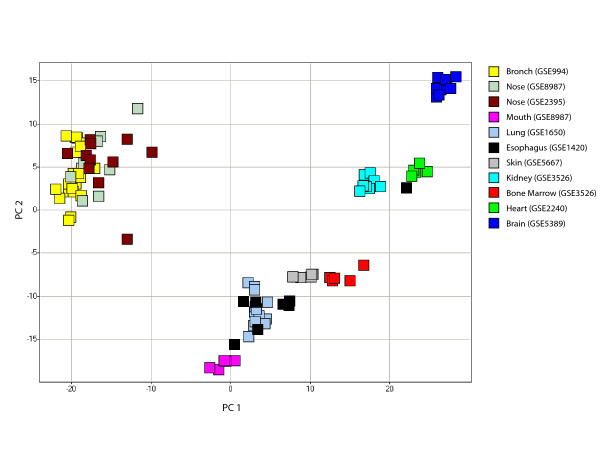
**Principal component analysis of 2382 genes from the normal bronchial airway transcriptome across 11 tissue datasets**. Principal component analysis (PCA) of the expression of 2382 genes from the never-smoker bronchial-airway transcriptome [8] in samples from 11 different tissue datasets. Bronchial and nasal epithelial samples group together along the first and second principal components (representing 66% and 12% of the variance in gene expression respectively) based on the expression of these genes. Tissue types are color-coded.

In order to explore gene-expression relationships between different airway epithelial tissues in healthy non smokers, principal component analysis (PCA) of the normal airway transcriptome [8] was performed across 11 different non-diseased tissue datasets (Figure 1). Nine microarray datasets containing normal tissue samples from previously published studies were collected from the Gene Expression Omnibus (GEO). These datasets were analyzed along with the 8 normal nasal epithelial and 5 normal buccal mucosa samples from this study. A detailed list of the different datasets used is shown in Table 2. Seven of the eleven datasets analyzed came from tissues of epithelial origin, including an additional set of normal nasal epithelial samples from a separate study [18]. The co-localization of bronchial and nasal samples in a graph of the first two principal components suggests a relationship between these two tissues when compared to other epithelial tissues analyzed. Buccal mucosa samples did not group with either bronchial or nasal epithelial samples.

**Table 2:** Description of all microarray datasets analyzed in this study.

**Category**	**Tissue**	**# Samples**	**Platform**	**GEO reference**	**Sample Description**
Epithelial	**Mouth**	5	U133A	GSE8987	5 never smokers
Epithelial	**Bronch**	23	U133A	GSE994	23 never smokers
Epithelial	**Nose**	8	U133A+2.0	GSE8987	8 never smokers
Epithelial	**Nose**	11	U133A	GSE2395	11 normal nasal epithelial samples; from cystic fibrosis study
Epithelial	**Lung**	14	U133A	GSE1650	no/mild emphysema patients; from COPD study
Epithelial	**Skin**	5	U133A	GSE5667	normal skin tissue
Epithelial	**Esophagus**	8	U133A	GSE1420	normal esophageal epithelium
Mostly epithelial	**Kidney**	8	U133+2.0	GSE3526	4 kidney cortex, 4 kidney medulla (post-mortem)
non epithelial	**Bone marrow**	5	U133+2.0	GSE3526	5 bone marrow (post-mortem)
non epithelial	**Heart**	5	U133A	GSE2240	left ventricular myocardium, non-failing
non epithelial	**Brain**	11	U133A	GSE5389	postmortem orbitofrontal cortex

Microarray datasets from 9 studies obtained from GEO, as well as the 2 datasets from this study are listed. Tissue type, total sample numbers, microarray platform, GEO accession number, and a brief description of each dataset are included.

**Figure 2:**
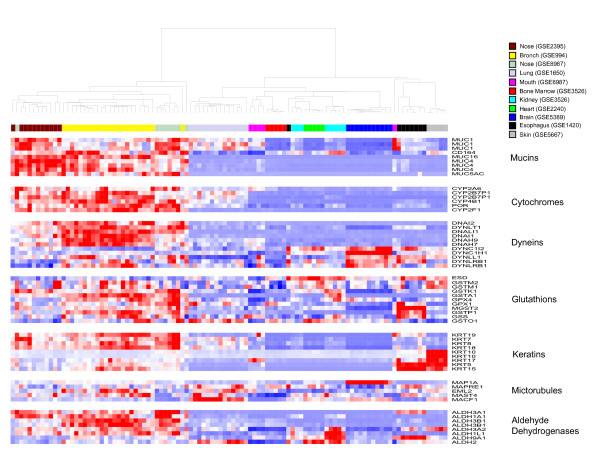
**Hierarchical clustering of 59 genes representing functional categories over-represented in the normal bronchial airway transcriptome**. Supervised hierarchical clustering of the expression of 59 probesets representing genes in the mucin, dynein/microtubule, cytochrome P450, glutathione, aldehyde dehydrogenase, and keratin functional categories across samples from 11 tissue datasets. Nasal and bronchial epithelial samples cluster together based on similar expression patterns across these groups of genes (high expression = red, average expression = white, low expression = blue).

To determine the similarities in the expression of functional categories of genes that are over represented in the never smoker bronchial airway transcriptome [8] and are likely to play a role in mediating the response to tobacco smoke exposure, we examined the expression of fifty-nine genes involved in detoxification (e.g. cytochrome P450 family, glutathiones, aldehyde dehydrogenases), as well as important epithelial cell structural components (e.g. mucins, dyneins, microtubule associated genes) across the 11 normal tissue datasets (Figure 2). Bronchial and nasal epithelial samples clustered together based on the expression of these 59 genes, with many being expressed at higher levels in these two tissues, including genes belonging to the dynein, cytochrome P450, and aldehyde dehydrogenase gene families. Buccal mucosa samples clustered with lung tissue, with specific keratin genes being highly expressed in both tissues. While some keratins were expressed specifically in skin and esophageal epithelium, other keratins, such as *KRT7*, *KRT8*, *KRT18*, and *KRT19 *were expressed primarily in bronchial and nasal samples. The same pattern was seen with mucin genes, with *MUC4*, *MUC5AC*, and *MUC16 *being expressed primarily in the bronchus and nose, while *MUC1 *was expressed in other epithelial tissues. Glutathione genes were expressed highly in bronchial and nasal epithelial tissue as well as other tissues.

### Effects of cigarette smoke on intrathoracic and extrathoracic epithelial gene expression

**Figure 3:**
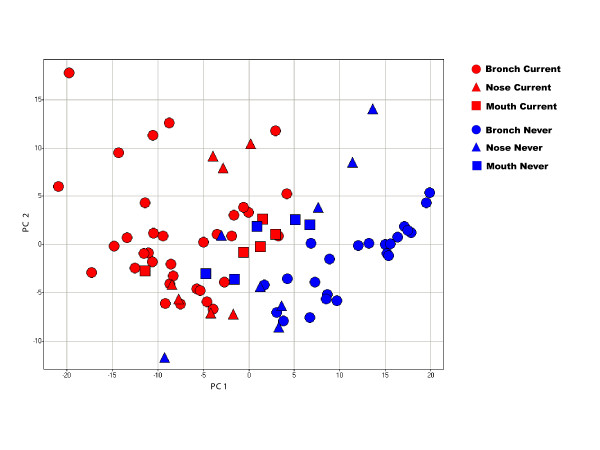
**Principal component analysis of 361 differentially expressed bronchial epithelial genes across bronchial, nasal, and buccal mucosa samples**. PCA of the expression of genes that are perturbed by smoking in the bronchial airway in samples of bronchial, nasal, and buccal epithelium from smokers and non-smokers. Bronchial and nasal epithelial samples separate according to smoking status, while the separation of buccal mucosa samples by smoking status is less pronounced. The first two principal components account for 27% and 9% of the total variance in gene expression respectively.

To examine the similarities of tobacco-induced differential gene expression between the bronchus and the buccal and nasal epithelium, gene expression profiles from buccal (n = 10) and nasal (n = 15) epithelial samples collected from current and never smokers were analyzed together with previously published bronchial epithelial samples collected from current and never smokers (n = 57) [8]. Three hundred and sixty-one genes differentially expressed (p < 0.001) between current and never smokers in bronchial epithelium [8] distinguish the bronchial and nasal epithelial samples by smoking status using principal component analysis, with separation among buccal mucosa samples being less clear (Figure 3). These results suggest that the gene-expression response to smoking is similar in bronchial and nasal epithelium.

**Figure 4:**
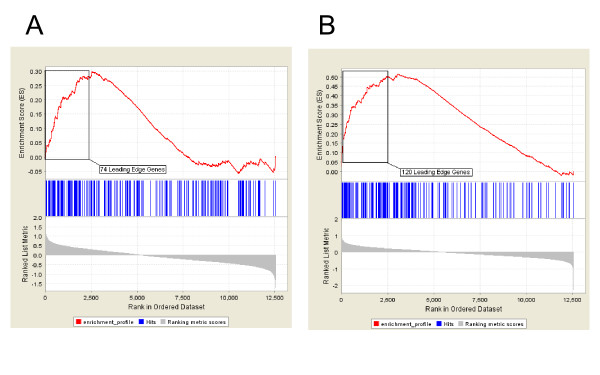
**Enrichment of differentially expressed bronchial epithelial genes among smoking-induced genes in the buccal mucosa or nasal epithelium**. Results from gene set enrichment analysis (GSEA) shows that 361 genes induced in bronchial epithelial cells from smokers are significantly skewed toward being among the genes most induced in buccal (A) or nasal (B) epithelial samples in response to smoking (p < 0.001). There are 74 genes that comprise the buccal "leading edge subset" and 120 genes that comprise the nasal "leading edge subset". These two analyses suggest that genes induced by smoking in the bronchus show a similar pattern of differential expression in buccal and nasal epithelium.

**Figure 5:**
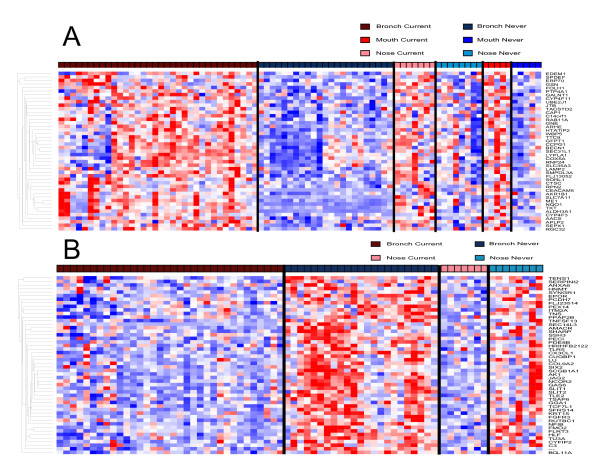
**Hierarchical clustering of genes commonly perturbed by smoking across intra- and extrathoracic airway epithelium**. A. Supervised hierarchical clustering of the expression of 45 genes induced by smoking in the bronchial airway that are present in both the nasal and buccal "leading edge subsets" in samples from smokers and non-smokers. These represent genes up-regulated by smoking in bronchial, nasal, and buccal epithelium. B. Supervised hierarchical clustering of the expression of 50 genes repressed by smoking in the bronchial airway that are present in the nasal "leading edge subset" in samples from smokers and nonsmokers. (High expression = red, average expression = white, low expression = blue).

Gene set enrichment analysis (GSEA) was performed (see Additional File 1) to determine which of the genes that are affected by smoking in bronchial epithelium are among the most highly perturbed by smoking in nasal and buccal epithelium. Genes up-regulated in the airway in response to smoking are significantly enriched among the genes most up-regulated by smoking in buccal mucosa (p < 0.001), with 74 genes composing the "leading edge subset" (Figure 4A). The leading edge consists of the subset of bronchial smoking-related genes that are most differentially expressed in response to smoking in buccal mucosa. Bronchial smoking-related genes are also significantly enriched among the genes that are differentially expressed in nasal epithelium in response to smoking (p < 0.001), with 120 genes comprising the leading edge subset (Figure 4B). Forty-five genes are common to both leading edges (Figure 5A, Additional File 5), suggesting that these genes represent common tobacco-induced changes that occur in all airway epithelial cells that are exposed to tobacco smoke. Genes with oxidoreductase and electron transporter activity are enriched among these commonly smoking-induced genes (p < 0.0001). Genes from the mainstem bronchus that are down-regulated in response to smoking were not significantly enriched among buccal mucosa genes most altered by smoking. However, down-regulated bronchial genes are enriched among genes most down-regulated in response to smoking in the nasal epithelium (p < 0.001), yielding a leading edge subset of 50 genes (Figure 5B). Genes involved in cell motility, cell migration, development, and localization of cellular complexes are enriched among these smoking-repressed genes (p < 0.001). In addition, we performed GSEA using the top 100 up- or 100 down-regulated-by-smoking genes from buccal and nasal epithelium and found that genes up-regulated in these tissues are enriched among the genes that are most perturbed by smoking in bronchial epithelium (see Additional File 1).

### Validation of buccal mucosa changes using real competitive PCR

Three genes from the buccal mucosa leading-edge subset were chosen for validation using real competitive PCR [19] (see Additional File 1) in an independent set of buccal epithelial samples obtained from 14 subjects (7 smokers, 7 never-smokers). Using a MALDI TOF mass spectrometry platform, gene expression analysis showed that all three genes (CEACAM5, CYP4F11, S100P) were more highly expressed in the oral epithelium of smokers compared with non-smokers, consistent with the microarray findings (Additional File 6).

## Discussion

We have studied global gene expression in bronchial, nasal, and buccal epithelial cells in never and current smokers. Our findings suggest that similar functional categories of genes are expressed in nasal and bronchial epithelial cells of healthy never smokers. We have shown that there are similarities between the effect of smoking on bronchial epithelial gene expression and the gene expression response to smoking in buccal and nasal epithelium. This implies the potential to study disease-relevant responses to tobacco smoke in any of these tissues. This represents a significant advantage as buccal and nasal mucosa can be readily collected from large study cohorts as a result of their ability to be collected non-invasively. Given the burden of smoking-related disease, there is a need for non-invasive biomarkers of the individual-level variability in host responses to cigarette smoke.

The similar pattern of gene expression in bronchial and nasal epithelial cells of never smokers suggests a shared architecture and function. The nasal passage and bronchus are both lined with ciliated pseudostratified columnar epithelial cells, and some of the functions of genes that are highly expressed in both nasal and bronchial epithelial cells are likely due to this common cellular architecture. For example, cilia-related genes such as *DNAH7*, *DNAH9*, and *DNAI2 *were highly expressed in both bronchial and nasal airway epithelium. Consistent with this finding, previous studies have shown that normal ciliated airway epithelial cells express these genes [20-22]. Other dynein light chain genes such as *DYNLRB1 *which have been characterized in other non-epithelial tissues [23] were found to be specifically expressed in bronchial epithelial cells, while dynein light chain genes such as *DYNLL1 *shared relatively high expression specifically in nasal epithelium as well as non-epithelial tissues. Many genes involved in host defense are also expressed at high levels in extra- and intrathoracic airway epithelial cells. Glutathione expression has been previously well characterized in bronchial epithelium as well as in the lung [24]. Our data show high expression of glutathiones such as *GSTM1 *in bronchial, nasal, and buccal epithelium relative to other non-airway epithelial cells. We also observed high expression of mucins such as *MUC1*, *MUC4*, and *MUC5AC *in bronchial and nasal epithelium and somewhat lower expression of *MUC1 *in buccal mucosa and lung tissue. Expression of these genes has been well documented in respiratory tract epithelium [25-29]. We found the genes belonging to the cytochrome P450 family and several aldehyde dehydrogenase genes [30] are highly expressed the bronchial and nasal epithelium. Cytochrome P450 genes have previously been shown to be expressed highly in both bronchial epithelial cells [31-33] and nasal mucosa [34]. Our global analysis of gene-expression of the airway epithelium of healthy never smokers recapitulates gene expression patterns previously observed within these tissue types, thereby lending support to the similarities and differences between tissues that are suggested by our analysis of gene expression in the normal intrathoracic airway transcriptome.

Smoking altered the expression of a set of genes in bronchial epithelial cells which were also commonly altered in nasal and buccal epithelial cells. Gene set enrichment analysis of 361 smoking-induced bronchial genes yielded a subset of genes that were among the most up-regulated by smoking in the mouth (74 leading edge genes), as well as a subset of genes that are among the most up-regulated by smoking in the nose (120 leading edge genes). Forty-five genes were common to both sets, representing genes that share similar smoking-related expression patterns across all three airway epithelial tissues. This analysis demonstrates a common response to cigarette smoke exposure in cells lining the respiratory tract. Within this set are multiple genes involved in each of several processes including detoxification, cell cycle progression and cell adhesion. In addition, a common set of genes was down-regulated in response to smoking in both bronchial and nasal epithelium.

Several CYP450 genes were commonly up-regulated by smoking in only the nose and airway including *CYP1A1 *and *CYP1B1*, as well as cell cycle (*CCNG2*, *RAB2*) and cell adhesion genes (*CEACAM5*, *CEACAM6*). The presence of mutations in *CYP1A1 *in nasal and bronchial mucosa has been shown previously in smokers [16], and cytochrome P450 genes are known to be involved in xenobiotic metabolism in bronchial mucosa [35]. Exposure of alveolar epithelial cells to environmental toxins has been shown to promote cell cycle progression [36], which could explain the increased expression of cell cycle genes in the nasal epithelial cells of smokers. Glutathiones such as *GPX2 *were up-regulated in both bronchial and buccal epithelial cells. Aldo-keto reducatse genes which are activated in response to cigarette smoke in human oral squamous cell lines [37], are also up-regulated in both bronchial and buccal epithelial samples. Oxidoreductase genes are up-regulated in all three airway epithelium including other CYP450 genes (*CYP4F3*, *CYP4F11*), aldehyde dehydrogenases (*ALDH3A1*), and aldo-keto reductases (*AKR1B1*), suggesting that smoking activates common detoxification pathways in exposed airway epithelial cells.

Gene set enrichment analysis also identified genes that are among the most down-regulated in both nasal and bronchial epithelium in response to tobacco exposure. *SLIT2*, which is a known tumor suppressor that is down-regulated in lung cancer [38,39] is among these genes. We found *HNMT*, which is expressed highly in both bronchial and nasal mucosa [40,41], and has been shown to be down-regulated with smoking in other mucosal cells [42], to be down regulated in smoking in bronchial and nasal epithelium. The genes that are among the most down regulated by smoking in both bronchial and nasal epithelium were enriched for those with functions in cellular localization, migration, and motility genes. These data suggest that smoking results in the down-regulation of structural genes in these tissues.

Based on the data presented here, we suggest that aspects of the bronchial gene expression response to smoking are also changed by smoking in nasal epithelium, with certain of these genes also being perturbed by smoking in buccal mucosa. This suggests that there are common features in the field of injury caused by cigarette smoke throughout the airway. Our data also suggest that the gene-expression consequences of smoking are less pronounced in buccal mucosa (see Figure 3). This could be due to a number of factors: 1) the effects of smoking on buccal mucosa might indeed be less pronounced; 2) there may be more inter-subject variability in buccal mucosa gene expression; 3) the partial degradation of the RNA in the buccal mucosa samples may contribute to variability in gene expression estimates. Due to the high concentration of RNAses found in saliva, RNA obtained from buccal epithelial cells was subject to degradation, and relatively small amounts of RNA were extracted from these cells. This required us to pool samples collected from the same individual serially over several weeks. Previous studies report similar issues with salivary RNA run on microarrays [43]. Consistent with the low yield of partially degraded RNA from buccal samples, we detected sequence-specific hybridization intensity for fewer probesets in the buccal samples than in the nasal or bronchial samples. Despite these technical limitations, there was considerable overlap between genes that are among the most altered in response to smoking in the nose and bronchus and those that are most differentially expressed in the mouth based on the overlap between the leading edge subsets from gene set enrichment analysis. Differential expression of several genes seen to be induced by smoking in buccal mucosa was validated in buccal mucosa samples from independent volunteers using real competitive PCR. Taken together, these findings indicate that gene expression is perturbed by smoking in buccal mucosa and suggest that techniques for assaying gene expression in the context of partially degraded RNA will facilitate further studies to determine if buccal-mucosa specific factors contribute to the apparent differences in the magnitude of the smoking response of buccal mucosa relative to that seen in other airway epithelia.

## Conclusion

This study highlights the relationships between gene expression profiles in epithelial cells that line the intra- and extra-thoracic airway and identifies a common set of genes that are induced by tobacco smoke in buccal, nasal and bronchial epithelium, supporting the concept that smoking induces a common field of injury throughout the airway. These similarities suggest that easily collected buccal and nasal epithelium can be used to measure an individual's physiologic response to tobacco smoke.

## Methods

### Study Population

We recruited current and never smoker volunteers from Boston Medical Center for buccal (n = 11) and nasal (n = 15) microarray studies, and subsequent buccal epithelial samples for real competitive PCR validation using mass spectrometry (n = 14). For each volunteer, a detailed smoking history was obtained including number of pack-years, number of packs per day, age started, and environmental tobacco exposure. Current smokers in each group had smoked at least 10 cigarettes per day in the past month, with at least a cumulative smoking history of 10 pack-years. Non-smoking volunteers with significant environmental cigarette exposure and subjects with respiratory symptoms, known respiratory, nasal or oral diseases, or regular use of inhaled medications were excluded. The study was approved by the Institutional Review Board of Boston Medical Center, and all subjects provided written informed consent.

### Buccal epithelial cell collection

Buccal epithelial cells were collected from 25 study participants as previously reported [10]. Briefly, we used a non-invasive method for obtaining small amounts of RNA from the mouth using a concave plastic tool with serrated edges. Using gentle pressure, the serrated edge was scraped 5 times against the buccal mucosa on the inside left cheek and placed immediately into 1 mL of RNA Later (Qiagen, Valencia, CA). The procedure was repeated for the inside right cheek and the cellular material was combined into one tube. After storage at room temperature for up to 24 hours, total RNA was isolated from the cell pellet using TRIzol reagent (Invitrogen, Carlsbad, CA) according to the manufacturer's protocol. The integrity of the RNA was assessed on a denaturing agarose gel. Epithelial cell content on a representative set of samples was quantified by cytocentrifugation at 700 × g (Cytospin, ThermoShandon, Pittsburgh, PA) of the cell pellet and staining with a cytokeratin antibody (Signet, Dedham, MA). Using this protocol, we were able to obtain an average of 1823 ng +/- 1243 ng of total RNA per collection. Buccal epithelial cells were collected serially over 6 weeks in order to obtain a minimum of 8 ug of RNA per subject for microarray analysis. For the 14 subjects included in the real competitive PCR validation, a single collection yielded sufficient RNA given the reduced requirement for starting material.

### Nasal epithelial cell collection

Using a nasal speculum (Bionix Medical Technologies, Toledo, OH), epithelial cells were collected from the right inferior turbinate with a standard cytology brush (Medical Packaging Company, Camarillo, CA). Brushings were immediately placed in RNA lysis buffer and snap frozen in liquid nitrogen. Samples were frozen at -80°C until use. RNA was isolated via Qiagen RNeasy Mini Kits per manufacturer's protocol. As above, the integrity of RNA was assessed with a denaturing agarose gel and epithelial cell content was quantified by cytokeratin staining of the cell pellet. We obtained an average of 25 ug of high-quality total RNA from a single collection.

### Microarray Data Acquisition and Preprocessing

Approximately eight micrograms of total RNA from buccal epithelial cells or nasal epithelial cells was processed, labelled, and hybridized to Affymetrix HG-U133A (buccal samples) or HG-U133A 2.0 (nasal samples) arrays each containing 22,215 probe sets as previously described [8]. A single weighted expression estimate for each probe set was derived using MICROARRAY SUITE 5.0 (MAS 5.0) software (Affymetrix, Santa Clara, CA). The MAS 5.0 software also generated a detection P value [P_(detection)_] using a one-sided Wilcoxon sign-ranked test, which indicates whether the expression estimate is significantly higher than that observed with single-base mismatch probes. Based on these detection P values, one buccal mucosa sample was excluded from further analysis as the percentage of genes detected in this sample was two standard deviations less than the median percentage detected across all buccal mucosa microarray samples. The remaining 10 buccal mucosa samples were analyzed further. Microarray data from 57 bronchial epithelial cell samples was obtained from a dataset (GSE994) previously published by our group [8].

Microarray data from 8 additional non-diseased human tissues were obtained from datasets in the Gene Expression Omnibus (GEO) as of September 2006. Non-diseased normal samples were selected from datasets where there were at least 5 samples per tissue type and CEL files were available for each sample so that all array data could be processed in MAS 5.0 in the same manner. All samples selected were run on either Affymetrix HGU133A or HGU133A 2.0 microarrays. Array data from normal tissue samples from the following 8 tissues were used (GEO accession number included): nose (GSE2395), lung (GSE1650), skin (GSE5667), esophagus (GSE1420), kidney (GSE3526), bone marrow (GSE3526), heart (GSE2240), and brain (GSE5389). Table 2 contains a detailed description of these datasets.

### Microarray Data Analysis – Exploring the normal airway transcriptome

To investigate the relationship between gene expression in airway epithelial tissues with respect to other epithelial and non-epithelial tissue types, 2382 genes expressed at detectable levels in the bronchial airway of healthy never smokers [8] were examined in microarray data from 11 normal tissue datasets (Table 2) using principal component analysis (PCA) performed using DecisionSite Software [44] (TIBCO Spotfire, Somerville, MA). Each dataset was first log transformed and subsequently z-score normalized within each sample within each dataset in order to minimize batch specific effects.

The expression of genes with functions relevant to airway epithelial cell biology was then analyzed across these samples to further explore the relationships among the tissues. Groups of genes involved in detoxification (cytochrome P450s, glutathiones, aldehyde dehydrogenases), as well as structural genes (mucins, dyneins, keratins) were selected based on overrepresentation of these functional categories among the never smoker bronchial airway transcriptome, resulting in a dataset of fifty-nine probesets. These were analyzed by supervised hierarchical clustering of samples using z-scored normalized data with a Pearson correlation (uncentered) similarity metric and average linkage clustering using CLUSTER and TREEVIEW software [45].

### Microarray Data Analysis – Effects of smoking on airway epithelium

We defined genes that are differentially expressed in the bronchial epithelium in response to tobacco smoke as those 361 probesets with a t-test p-value less than 0.001 when comparing smokers to non-smokers from our previously published dataset [8]. This set of genes that is perturbed by smoking in bronchial epithelium was used to explore the effects of smoking in samples of nasal and buccal mucosa from smokers and non-smokers. PCA was performed across 82 smoker and non-smoker samples (57 bronchial, 10 buccal, 15 nasal) using the 361 probesets (corresponding to 314 unique genes) described above. The distribution of these genes within the list of all genes ranked according to the degree of perturbation by smoking in either buccal and nasal epithelial samples was also assessed using gene set enrichment analysis (GSEA) [46]. For each tissue, genes were ranked from most induced by smoking to most repressed by smoking using the signal-to-noise ratio for the effect of smoking. Empiric P-values for the skewness of the observed distributions were generated in GSEA by permuting the gene labels. A significant p-value in this analysis indicates that the bronchial airway smoking-related genes tend to either be induced or repressed within the samples being analyzed. This analysis also yielded enriched gene sets, or "leading edge subsets" which represent the bronchial airway smoking-related genes that contribute most to the observed uneven distribution. A detailed flow of the GSEA performed can be found in Additional Files 2, 3. Supervised hierarchical clustering of genes was performed using z-scored normalized data with a Pearson correlation (uncentered) similarity metric and average linkage clustering using CLUSTER and TREEVIEW software to visualize the expression of genes common to both leading edge subsets.

### Validation by real competitive PCR using mass spectrometry

The differential expression of three out of the 74 genes from GSEA of smoking-induced bronchial genes perturbed in buccal mucosa samples (Figure 4A) was analyzed in fourteen additional buccal mucosa specimens using real-time competitive PCR [19] (see Additional File 1).

## Availability and requirements

All statistical analyses described were performed using R v. 2.2.0 [47]. The gene annotations used for each probe set were from the December 2004 NetAffx HG-U133A annotation file. Additional information from this study, including expression levels from all genes in all samples, and relevant clinical data on all subjects are available from our interactive database  which supports user-defined statistical and graphical analyses of data. Data from nasal and buccal epithelial microarray experiments have also been submitted to the National Center for Biotechnology Information Gene Expression Omnibus (GSE8987).

## Authors' contributions

SS contributed to the design of the analytic strategy and was responsible for the computational analysis and its interpretation. FS contributed to the gene-expression analysis, performed the mass spectrometry gene-expression assays, and contributed to analysis of mass-spectrometry data. JZ AMG and VS contributed to gene-expression analysis of non-diseased tissue datasets. KS contributed to study coordination and analysis of gene-expression data. GL and XZ performed the buccal and nasal microarray experiments and GL contributed to running mass spectrometry assays. YMD was responsible for coordinating all patient recruitment and sample collection. JB contributed to the design of the study and biological interpretation of the data. MEL conceptualized aspects of the analytic strategy. AS was responsible for the conception and design of this study and oversaw all aspects of the study including patient recruitment, experimental protocols and data analysis. All authors have read and approved the final manuscript.

## Supplementary Material

Additional File 1 Additional Methods. Information provided represents further methodology for Gene Set Enrichment Analysis comparing expression of genes in buccal, nasal, and bronchial epithelial datasets, as well as methodology for real-competitive PCR analysis of buccal mucosa gene expression. Click here for file

Additional File 2 Gene Set Enrichment Analysis Strategy. Strategic flow for Gene Set Enrichment Analysis to determine the distribution of genes differentially expressed in the bronchial epithelium of smokers within the ranked list of gene expression differences observed between smokers and non-smokers in buccal and nasal epithelial samples. Click here for file

Additional File 3 Figure legend for Gene Set Enrichment Analysis Strategy. Information provided represents the figure legend for Additional Figure 1, explaining the strategic flow of gene set enrichment analysis described in the main manuscript. Click here for file

Additional File 4 Subject demographics for real competitive PCR studies. Data provided represents demographics for buccal mucosa samples used in the real competitive PCR studies. Click here for file

Additional File 5 Differential expression of overlapping leading edge genes. Data provided represents fold change and p-values for the 5 most differentially expressed genes in the nose or mouth among the 45 genes induced by smoking in the bronchial airway that are present in both the nasal and buccal "leading edge subsets" Click here for file

Additional File 6 Real competitive PCR results. Data provided represents gene expression and fold change for three genes validated in additional buccal mucosa samples from Additional File 4 via real competitive PCR. Click here for file
