# Supplementary material for: Smoking-induced gene expression changes in the bronchial airway are reflected in nasal and buccal epithelium
Source: BMC Genomics. 2008 May 30;9:259. doi: 10.1186/1471-2164-9-259 (PMC2435556; doi:10.1186/1471-2164-9-259)
Supplement: Additional File 1 — Additional Methods. Information provided represents further methodology for Gene Set Enrichment Analysis comparing expression of genes in buccal, nasal, and bronchial epithelial datasets, as well as methodology for real-competitive PCR analysis of buccal mucosa gene expression. [file 1471-2164-9-259-S1.doc]

# Additional File 1

**Determining the effects of cigarette smoke on intrathoracic and extrathoracic epithelial gene expression**

Gene set enrichment analysis (GSEA) [1] was used to determine the distribution of 361 probesets previously identified to be differentially expressed between smokers and non-smokers in bronchial epithelial cells [2] within the ranked list of gene expression differences observed between smokers and non-smokers in buccal and nasal epithelial samples. The 361 probesets corresponded to 314 unique genes, 206 of which were up-regulated in smokers while 108 were down-regulated. GSEA was first used to analyze the distribution of the 206 genes up-regulated in the bronchial epithelium of smokers within the ranked list of the gene expression differences observed in buccal (n = 10, 5 current smokers, 5 never smokers) and nasal (n = 15, 7 current smokers, 8 never smokers) epithelial samples, where the genes were ranked from most induced by smoking to most repressed by smoking by a signal-to-noise ratio. Empiric p-values for the skewness of the observed distributions were generated in GSEA by permuting the gene labels. A significant p-value indicated that the up-regulated smoking-induced bronchial genes as a set also changed in the same direction in nasal or buccal mucosa of smokers. This analysis also yielded “leading edge subsets” which represent the bronchial airway smoking-related genes that contribute most to the observed uneven distribution. This analysis was repeated to determine the distribution of the 108 bronchial genes down-regulated by smoking among genes down-regulated by smoking in nasal and buccal mucosa samples. The strategy for this analysis is shown in Additional Figure 1.

In addition, we performed GSEA using the top 100 up- or 100 down-regulated-by-smoking genes from buccal and nasal epithelium among the ranked list of gene expression differences observed between smokers and non-smokers in the bronchus (n = 57, 34 current smokers, 23 never smokers). Our goal was to determine if genes which change in buccal or nasal epithelial cells in response to smoking are also differentially expressed in the intrathoracic airway in response to smoking. We found that genes up-regulated in the nasal epithelium or buccal mucosa of smokers were each enriched among the set of genes that were up-regulated in the bronchial airways of smokers (FDR < 0.01, FDR < 0.15 respectively). FDR values were generated by permuting class labels within the bronchial epithelial dataset, as is recommended for larger datasets. In addition, genes which were down-regulated in nasal epithelium in response to smoking were enriched among genes down-regulated in the bronchial epithelium of smokers (FDR < 0.01).

**Real competitive PCR using mass spectrometry**

The induction of three genes -- CEACAM5, CYP4F11 and S100P -- up-regulated by smoking in bronchial epithelium and found in the buccal smoking-induced leading edge subset (Figure 4A) was tested in fourteen additional buccal mucosa specimens using real competitive PCR as previously described [3]. The demographic information for these 14 subjects is shown in Additional Table 1. One of these three genes (CYP4F11) was also among the 45 leading-edge genes (Figure 5A, Additional Table 2) commonly up-regulated across bronchial, nasal, and buccal epithelium. We chose to validate these three genes based on our interest in their biological function. They are involved in pathways including cellular adhesion, cellular migration, calcium ion binding, electron transport and ion binding, which play a role in epithelial cell response to oxidant stress.

Approximately 25ng of starting RNA was used for a random-hexamer primed reverse transcription. A known concentration of an 80 bp oligonucleotide ‘competitor’(IDT, Indiana) differing from the gene of interest (‘wild type’) by only one base pair was then added to each sample. A subsequent PCR reaction was done which amplified both the competitor and the gene of interest. After a base extension reaction, the PCR products were spotted on a 384-format silicon chip and quantified via mass spectrometry. By comparing the area under the mass spectrometry peaks for the ‘competitor’ and ‘wild type’ products, absolute gene expression quantification was derived. A set of 10 housekeeping genes run on each sample as a single multiplex allowed geometric mean normalization with the most stable genes [4]. Gene expression among the 3 genes run in a second multiplex was then compared between the groups of smokers and non-smokers in the buccal epithelium (n=14, 7 smokers, 7 never-smokers). All three genes (CEACAM5, CYP4F11 S100P) were up-regulated in the oral epithelium of smokers compared with non-smokers, consistent with the microarray findings, as shown in Additional Table 3.

# References

# 1. Subramanian A, Tamayo P, Mootha VK, Mukherjee S, Ebert BL, Gillette MA, Paulovich A, Pomeroy SL, Golub TR, Lander ES *et al*: Gene set enrichment analysis: a knowledge-based approach for interpreting genome-wide expression profiles. *Proc Natl Acad Sci U S A* 2005, 102(43):15545-15550.

# 2. Spira A, Beane J, Shah V, Liu G, Schembri F, Yang X, Palma J, Brody JS: Effects of cigarette smoke on the human airway epithelial cell transcriptome. *Proc Natl Acad Sci U S A* 2004, 101(27):10143-10148.

# 3. Ding C, Cantor CR: A high-throughput gene expression analysis technique using competitive PCR and matrix-assisted laser desorption ionization time-of-flight MS. *Proc Natl Acad Sci U S A* 2003, 100(6):3059-3064.

# 4. Vandesompele J, De Preter K, Pattyn F, Poppe B, Van Roy N, De Paepe A, Speleman F: Accurate normalization of real-time quantitative RT-PCR data by geometric averaging of multiple internal control genes. *Genome Biol* 2002, 3(7):RESEARCH0034.
