# Supplementary material for: Smoking-induced gene expression changes in the bronchial airway are reflected in nasal and buccal epithelium
Source: BMC Genomics. 2008 May 30;9:259. doi: 10.1186/1471-2164-9-259 (PMC2435556; doi:10.1186/1471-2164-9-259)
Supplement: Additional File 2 — Gene Set Enrichment Analysis Strategy. Strategic flow for Gene Set Enrichment Analysis to determine the distribution of genes differentially expressed in the bronchial epithelium of smokers within the ranked list of gene expression differences observed between smokers and non-smokers in buccal and nasal epithelial samples. [file 1471-2164-9-259-S2.pdf]

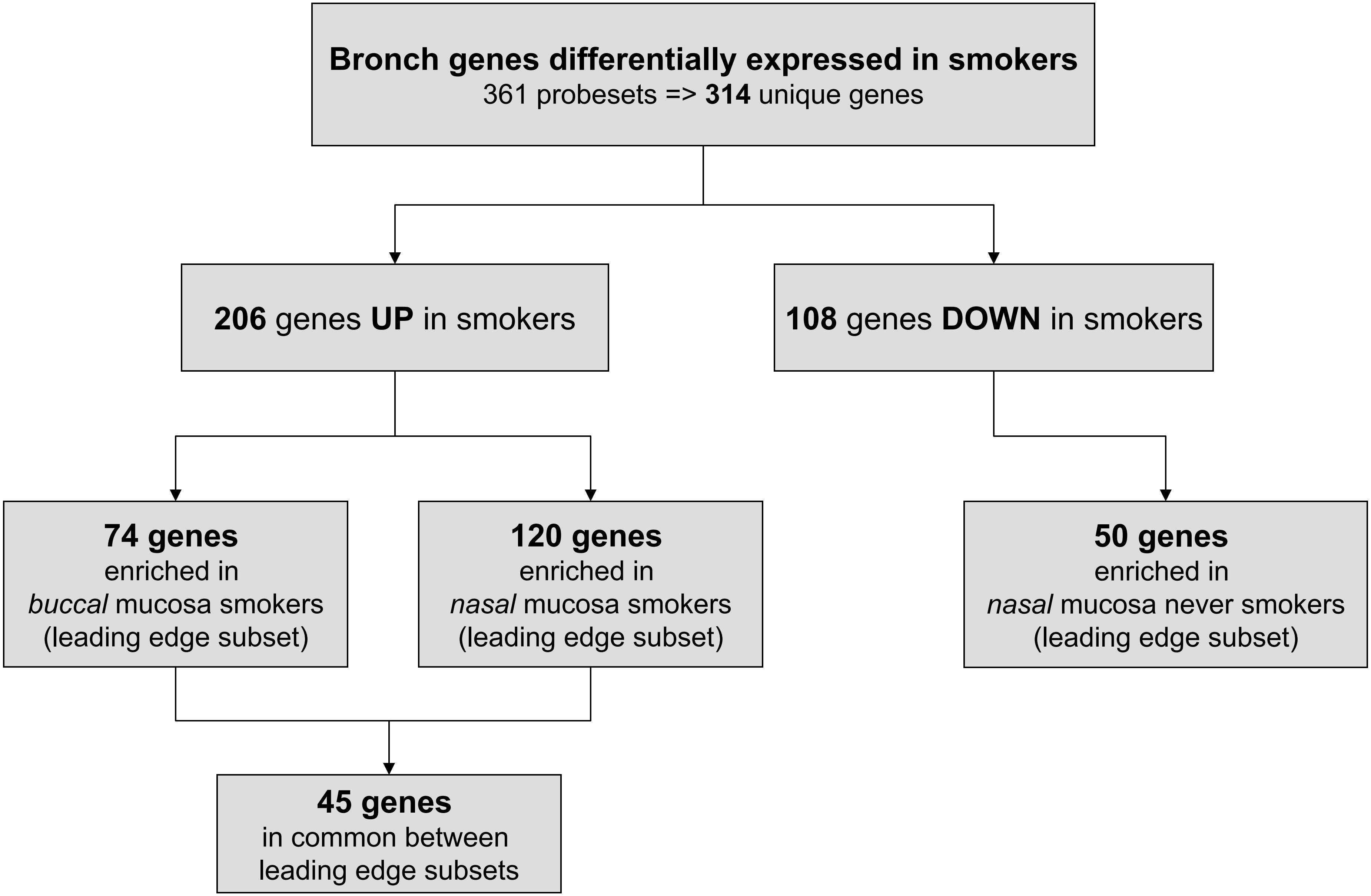
Bronch genes differentially expressed in smokers
361 probesets => **314** unique genes
206 genes **UP** in smokers
108 genes **DOWN** in smokers
74 genes
enriched in
buccal mucosa smokers
(leading edge subset)
120 genes
enriched in
nasal mucosa smokers
(leading edge subset)
50 genes
enriched in
nasal mucosa never smokers
(leading edge subset)
45 genes
in common between
leading edge subsets
