# Supplementary material for: Smoking-induced gene expression changes in the bronchial airway are reflected in nasal and buccal epithelium
Source: BMC Genomics. 2008 May 30;9:259. doi: 10.1186/1471-2164-9-259 (PMC2435556; doi:10.1186/1471-2164-9-259)
Supplement: Additional File 3 — Figure legend for Gene Set Enrichment Analysis Strategy. Information provided represents the figure legend for Additional Figure 1, explaining the strategic flow of gene set enrichment analysis described in the main manuscript. [file 1471-2164-9-259-S3.doc]

# Additional File 3

**Additional Figure 1 – Gene Set Enrichment Analysis flow**

Algorithm used for Gene Set Enrichment Analysis. Three hundred and sixty one bronchial genes were divided based on direction of expression in smokers and non-smokers, based on a previous study [2]. There was significant enrichment of the set of bronchial genes up-regulated by smoking in both the nasal and buccal mucosa of smokers. There was also significant enrichment of genes down-regulated by smoking in the bronchus in the nasal mucosa of never smokers. Genes down-regulated in the bronchial epithelium of smokers were not over-represented among genes down-regulated in the buccal mucosa of smokers.
