# Supplementary material for: Smoking-induced gene expression changes in the bronchial airway are reflected in nasal and buccal epithelium
Source: BMC Genomics. 2008 May 30;9:259. doi: 10.1186/1471-2164-9-259 (PMC2435556; doi:10.1186/1471-2164-9-259)
Supplement: Additional File 4 — Subject demographics for real competitive PCR studies. Data provided represents demographics for buccal mucosa samples used in the real competitive PCR studies. [file 1471-2164-9-259-S4.doc]

# Additional File 4

**Additional Table 1 –Subject demographics for real competitive PCR studies**

|  | **Current Smokers (n = 7)** | **Never Smokers (n = 7)** | **P-Value** |
| --- | --- | --- | --- |
| Sex | 6 M, 1 F | 4 M, 3 F | p = 0.24 |
|
| Age | 59 (+/- 15) | 41 (+/- 17) | p = 0.06 |
|
| Race | 5 CAU, 2 AFA | 4 CAU, 3 AFA | p = 0.37 |

Demographic data for mouth samples used in the real competitive PCR studies (n = 14). Clinical information including sex, age, and race are included (CAU – Caucasian, AFA – African American, HIS – Hispanic). P-values for sex and race were calculated by Fisher Exact test.
