# Supplementary material for: Smoking-induced gene expression changes in the bronchial airway are reflected in nasal and buccal epithelium
Source: BMC Genomics. 2008 May 30;9:259. doi: 10.1186/1471-2164-9-259 (PMC2435556; doi:10.1186/1471-2164-9-259)
Supplement: Additional File 5 — Differential expression of overlapping leading edge genes. Data provided represents fold change and p-values for the 5 most differentially expressed genes in the nose or mouth among the 45 genes induced by smoking in the bronchial airway that are present in both the nasal and buccal "leading edge subsets" [file 1471-2164-9-259-S5.doc]

# Additional File 5

**Additional Table 2 – Differential expression of overlapping leading edge genes.**

A.

| **GeneName** | **Fold Change (Bronch)** | **p-value (Bronch)** | **Fold Change (Nose)** | **p-value (Nose)** |
| --- | --- | --- | --- | --- |
| ALDH3A1 | 7.1 | 2.9E-10 | 1.5 | 4.7E-03 |
| TTC9 | 1.5 | 5.3E-04 | 1.3 | 1.1E-02 |
| FOLH1 | 1.6 | 8.8E-05 | 1.7 | 1.2E-02 |
| PDIA4 | 1.3 | 7.1E-04 | 1.3 | 1.8E-02 |
| TMED2 | 1.4 | 1.8E-04 | 1.2 | 1.9E-02 |

B.

| **GeneName** | **Fold Change (Bronch)** | **p-value (Bronch)** | **Fold Change (Mouth)** | **p-value (Mouth)** |
| --- | --- | --- | --- | --- |
| PTP4A1 | 1.5 | 5.3E-05 | 2.1 | 7.5E-04 |
| GNE | 1.6 | 1.0E-04 | 2.0 | 2.0E-02 |
| LAMP2 | 1.3 | 9.2E-04 | 1.4 | 3.6E-02 |
| JTB | 1.2 | 4.4E-04 | 1.2 | 4.1E-02 |
| GALNT1 | 1.3 | 4.3E-05 | 1.5 | 6.1E-02 |

Fold change and p-values for the 5 most differentially expressed genes in the (A) nose or (B) mouth among the 45 genes induced by smoking in the bronchial airway that are present in both the nasal and buccal “leading edge subsets”. P-values were calculated using a student’s t-test across all smoker and non-smoker samples within each tissue type. Fold-changes were calculated using a ratio of the average expression of smokers vs. non-smokers.
