# Supplementary material for: Smoking-induced gene expression changes in the bronchial airway are reflected in nasal and buccal epithelium
Source: BMC Genomics. 2008 May 30;9:259. doi: 10.1186/1471-2164-9-259 (PMC2435556; doi:10.1186/1471-2164-9-259)
Supplement: Additional File 6 — Real competitive PCR results. Data provided represents gene expression and fold change for three genes validated in additional buccal mucosa samples from Additional File 4 via real competitive PCR. [file 1471-2164-9-259-S6.doc]

# Additional File 6

**Additional Table 3 – Real competitive PCR results**

| **Gene** | **Smokers**  **(gene expression [M])** | **Nonsmokers**  **(gene expression [M])** | **Fold Change (Current/Never)** |
| --- | --- | --- | --- |
| CEACAM5 | 2.1E-15 | 1.5E-15 | 1.4 |
| CYP1F11 | 5.4E-17 | 1.7E-17 | 3.1 |
| S100P | 1.2E-15 | 4.1E-16 | 3.0 |

Gene expression (measured in average concentration [M] per group) and fold change for three genes validated in additional buccal mucosa samples from Additional Table 1 via real competitive PCR. The three genes were among the 74 gene leading edge subset of those up-regulated with smoking in both bronchial and buccal epithelium (Figure 4A). Expression of these genes changes in the same direction as seen in the buccal mucosa microarray studies.
